# Synthesis, computational studies and assessment of *in vitro* inhibitory activity of umbelliferon-based compounds against tumour-associated carbonic anhydrase isoforms IX and XII

**DOI:** 10.1080/14756366.2020.1786821

**Published:** 2020-07-02

**Authors:** Francesca Mancuso, Laura De Luca, Andrea Angeli, Sonia Del Prete, Clemente Capasso, Claudiu T. Supuran, Rosaria Gitto

**Affiliations:** a Dipartimento di Scienze Chimiche, Biologiche, Farmaceutiche ed Ambientali (CHIBIOFARAM), Università degli Studi di Messina, Messina, Italy; b Dipartimento NEUROFARBA, Università di Firenze, Sesto Fiorentino, Italy; c Istituto di Bioscienze e Biorisorse – CNR, Napoli, Italy

**Keywords:** Carbonic anhydrase inhibitors (CAIs), tumour-associated CA isoforms, coumarin, Pechmann condensation, Fries rearrangement

## Abstract

Coumarins are widely diffused secondary metabolites possessing a plethora of biological activities. It has been established that coumarins represent a peculiar class of human carbonic anhydrase (hCA) inhibitors having a distinct mechanism of action involving a non-classical binding with amino acid residues paving the entrance of hCA catalytic site. Herein, we report the synthesis of a small series of new coumarin derivatives **7-11**, **15**, **17** prepared via classical Pechmann condensation starting from resorcinol derivatives and suitable β-ketoesters. The evaluation of inhibitory activity revealed that these compounds possessed nanomolar affinity and high selectivity towards tumour-associated hCA IX and XII over cytosolic hCA I and hCA II isoforms. To investigate the binding mode of these new coumarin-inspired inhibitors, the most active compounds **10** and **17** were docked within hCA XII catalytic cleft.

## Introduction

**Figure 1.**
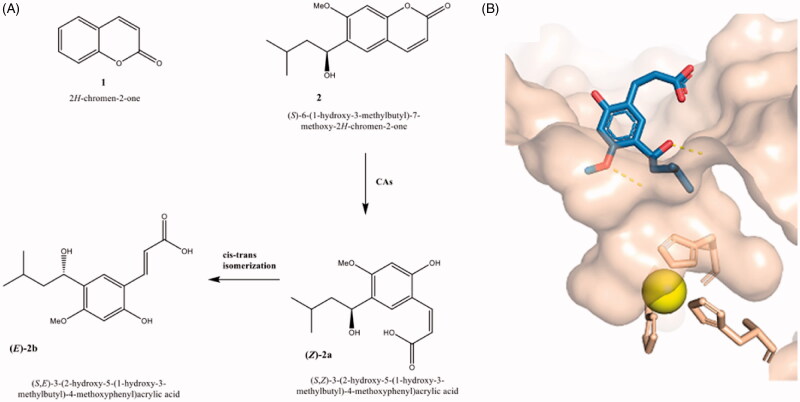
(A) Chemical structures of coumarin **1,** 6-(1-*S*-hydroxy-3methylbutyl)-7-methoxy-2*H*-chromen-2-one (**2**) and corresponding hydrolytic products **2a** and **2b**; (B) cocrystal structure of hydrolytic product of 6-(1-*S*-hydroxy-3methylbutyl)-7-methoxy-2*H*-chromen-2-one (**2**) in complex with hCA II (PDB code: 3F8E)^12^.

Coumarin (2*H*-chromen-2-one **1**, Figure 1) is classified as a member of a large class of phenolic chemotypes occurring in higher plants, bacteria and fungi. Apart from natural coumarins, a wide collection of (semi)synthetic coumarins are endowed by various pharmacological properties such as anticancer, anticoagulant, anti-inflammatory, and antimicrobial activities^1^. From a structural point of view, the coumarins are classified in different chemical classes (simple coumarins, fused polycyclic coumarins, phenylcoumarins, biscoumarins, and so on)^1^^,^^2^. The existence of an extensive library of coumarins from (semi)synthetic source has been triggered by the high versatility of benzo-α-pyrone system to be an excellent scaffold to perform structural modifications^3–6^. Moreover, the chemical diversity of the coumarin system implies that they might play an important role in medicinal chemistry for drug discovery processes, so that many coumarins are used currently in drug development as vitamin K antagonists, choleretic and antibacterial and antiviral agents^7–11^. It has been suggested that the benzopyrone structure enables its derivatives to readily interact with biological molecular targets through a network of non-covalent interactions such as hydrophobic, π–π stacking, and cation–π interactions as well as hydrogen and coordination bond interactions. Specifically, coumarin-based compounds interact with human carbonic anhydrases (CAs, EC 4.2.1.1), which possess significant esterase activity and induce the opening of lactone ring; due to the formation of the corresponding carboxylic derivative, non-classical CA inhibitory effects have been evidenced for coumarin-derived compounds (*vide infra*)^12–14^.

Human Carbonic anhydrases (hCAs) are a family of metalloenzymes belonging to α-class and comprising 12 catalytic isoforms (cytosolic CA I, CA II, CA III, CA VII and CA XIII; membrane-associated CA IV, CA IX, CA XII, and CA XIV; mitochondria-associated CA VA and CA VB; and secreted CA VI) that differ for kinetic properties, tissue distribution and subcellular localisation^15^^,^^16^. They catalyse the simple reaction of CO_2_ hydration leading to the formation of bicarbonate and proton. This reaction is a crucial physiological process for different organisms in controlling pH as well as relevant metabolic pathway. It has emerged that the CA-mediated pH regulation can offer the opportunity to develop therapeutics able to inhibit the CA activity related to pathological processes^16–18^. Among them, there is growing evidence that the two membrane-bound hCA IX and hCA XII isoforms might cause the extracellular pH of tumour cell microenvironment (TME) to be acid, thus playing a key role in the cancer progression and metastatic processes^19^. The dimeric hCA IX isoform is over-expressed in hypoxic tumour cells, modulated by the hypoxia-inducible factor (HIF-1), and results typically associated with aggressive tumour not responsive to chemotherapy and radiotherapy^20^. In tandem with the hCA IX isoform, the cancer-related hCA XII is involved in TME regulation; for this reason, oncogenic hCA IX/XII isoforms represent promising antitumor targets for the development of selective CA inhibitors able to reduce the growth of hypoxic cancers. The proof-of-concept that hCA IX inhibition is a profitable target has been confirmed by the therapeutically effective inhibitor SLC-0111, which has recently entered clinical trials for breast and brain cancer^21–28^.

Several coumarin-based compounds demonstrated to be potent hCA IX/XII inhibitors having a non-classical mechanism of CA-inhibition by occlusion of the enzymatic site^11^^,^^29–34^ that displays a cone-shape cavity containing a zinc ion located in the bottom and coordinated by three crucial histidine residues (His94, His96, and His119). Their unusual binding mode has been demonstrated for the coumarins such as the 6-(1-*S*-hydroxy-3methylbutyl)-7-methoxy-2*H*-chromen-2-one (**2**) through X-ray crystallography for the corresponding hydrolytic product (see Figure 1(B), PDB code: 3F8E)^12^; in detail, these studies evidenced a hydrolytic pathway that led to the hydrolytic product 2-hydroxycinnamic acid derivative bound at the entrance of the enzyme pocket hindering the access of substrate to the catalytic active site and blocking the catalytic activity.

**Figure 2.**
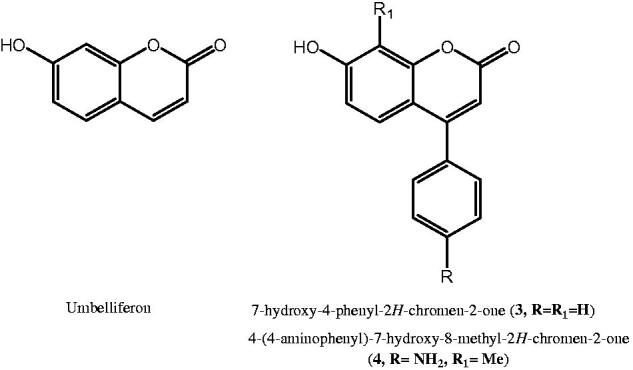
Chemical structures of umbelliferon, **3** and **4** as coumarin-based hCAIs.

As a result, the coumarin-based compounds might bind the most variable region located in the top area of the protein cavity for twelve catalytic isoforms, explaining the high selectivity towards specific druggable isoforms such as hCA IX/XII in combination with low affinity for the ubiquitous hCA I and II. Based on this evidence, we have recently synthesised a series of 4-phenyl-coumarin derivatives (e.g. **3** and **4,** Figure 2) and structurally related to umbelliferon (7-hydroxy-2H-chromen-2-one, Figure 2) which represents a selective and potent CA inhibitor^29^; notably, some of the new synthesised derivatives exhibited high affinity towards hCA IX and hCA XII with *K*_i_ values in the low nanomolar range; additionally, similar to other well-known coumarin-based compounds, they were inactive towards the ubiquitous hCA I and hCA II up to 10 µM concentration^29^. By using the best active and selective inhibitors 7-hydroxy-4-phenyl-2*H*-chromen-2-one (**3**, R = R_1_=H) and 4-(4-aminophenyl)-7-hydroxy-8-methyl-2*H*-chromen-2-one (**4**, R = NH_2_, R_1_=Me) as templates, we now report the synthesis of a further series of coumarins, which were tested as inhibitors of selected hCA I, II, IX and XII isoforms. Moreover, docking simulations analysed the possible interactions within the catalytic cavity of hCA XII^29^.

## Material and methods

### Chemistry

All reagents were used without further purification and bought from common commercial suppliers. Melting points were determined on a Buchi B-545 apparatus (BUCHI Labortechnik AG Flawil, Switzerland) and are uncorrected. By combustion analysis (C, H, N) carried out on a Carlo Erba Model 1106-Elemental Analyser, we determined the purity of synthesised compounds; the results confirmed a  ≥ 95% purity. Merck Silica Gel 60 F254 plates were used for analytical TLC (Merck KGaA, Darmstadt, Germany). For detection, iodine vapour and UV light (254 nm) were used. ^1^H NMR and ^13^C NMR spectra were measured in dimethylsulfoxide-d6 (DMSO-d_6_) with a Varian Gemini 300 or 500 spectrometer (Varian Inc. Palo Alto, California USA); chemical shifts are expressed in δ (ppm) and coupling constants (*J*) in hertz. All exchangeable protons were confirmed by the addition of D_2_O. R*_f_* values were determined on TLC plates using a mixture of CycloEx/EtOAc (60:40 v/v) as eluent. For coumarins **7–11** and **15**, the CAS registry numbers have been already assigned; however detailed information about chemical characterisation is not available in the literature; for selected compounds, representative ^1^H-NMR and ^13^C-NMR spectra are displayed in Supporting Material. Pharmacokinetics and drug-likeness prediction for the synthesised compounds (**7-11**, **15** and **17**) were performed by using the online tool SwissADME of Swiss Institute Bioinformatics (http://www.sib.swiss) and the collected data are shown in Supplemental Material.

#### Synthesis of 2-oxo-4-phenyl-2H-chromen-7-yl acetate (7)

To an ice-cold solution of resorcinol (**6**, 1 mmol) in the appropriate ethyl benzoylacetate derivative (**5**, 1 mmol), 96% w/v sulphuric acid (2 ml) was added dropwise. The mixture was brought to room temperature and stirred at 350 rpm by a stirring magnet bar for 24 h, then TLC showed the disappearance of both starting materials. The reaction mixture was quenched with crushed ice flakes, subsequently diluted with H_2_O (10 ml) and extracted with EtOAc (3 × 10 ml). The organic layer was dried with Na_2_SO_4_ and concentrated until dryness under reduced pressure. The targeted compounds **3** was isolated from the crude by crystallisation with EtOH. The spectroscopic data of compound **3** were consistent with those previously reported in the literature^29^. Subsequently, compound **3** (1 mmol) was stirred with acetic anhydride (3 ml) in an ice bath and a catalytic amount of 96% sulphuric acid was added dropwise. Then, Et_3_N (2.5 molar equivalents) was added to the mixture and stirred until the disappearance of starting compounds (TLC). After the reaction was completed, it was quenched with ice and the solid was filtered off and dried to afford the corresponding desired compound **7** (CAS Number: 16299-27-7) for which the structural assignments were in good agreement with the literature^35^.

Yield: 79%; m.p.: 129–131 °C; R*_f_* 0.64; ^1^H-NMR (CDCl_3_) (*δ*): 2.32 (s, 3H, CH_3_) 6.44 (s, 1H, CH), 7.17 (m, 1H, ArH), 7.40 (s, 1H, ArH), 7.48 (m, 1H, ArH), 7.55-7.60 (m, 5H, ArH). Anal. for (C_17_H_12_O_4_): C 72.85%, H 4.32%; Found: C 72.88%, H 4.45%.

### General synthesis for 2-oxo-4-phenyl-2H-chromen-7-yl propionate (8) and 2-oxo-4-phenyl-2H-chromen-7-yl benzoate (9)

To a stirred solution of compound **3** (1 mmol) in DCM (2 ml) and Et_3_N (2.5 molar equivalents), propionyl, or benzoyl chloride (5 molar equivalents) were slowly added in an ice bath. After 10 min, the mixture was allowed to room temperature and stirred until the complete conversion of starting material (TLC). Then, the solid products were filtered off and recrystallized from Et_2_O. For compounds **8** and **9**, detailed structural assignments are not available in the literature.

**2-Oxo-4-phenyl-2H-chromen-7-yl propionate (8)** (CAS Number: 327048-31-7)

Yield: 20%; m.p.: 172–174 °C; *R*_f_ 0.80; ^1^H-NMR (DMSO-d_6_) (*δ*): 1.16 (t, *J* = 7.3, 3H, CH_3_), 2.67 (q, *J* = 7.3, 2H, CH_2_), 6.43 (s, 1H, CH), 7.17 (d, *J* = 8.4, 2H, ArH), 7.40 (s, 1H, ArH), 7.48 (d, *J* = 8.4, 2H, ArH), 7.55–7.63 (m, 5H, ArH). ^13^C NMR (DMSO-d_6_) (*δ*): 8.77, 26.99, 110.71, 114.22. 116.37, 118.71, 127.82, 128.58, 128.99, 129.85. 134.71, 153.17, 154.26, 154.64, 159.56, 172.20. Anal. For (C_18_H_14_O_4_): C 73.46%, H 4.79%. Found: C 73.54%, H 4.77%.

**2-Oxo-4-phenyl-2H-chromen-7-yl benzoate (9)** (CAS Number: 94739-93)

Yield: 98%; m.p. 228–230 °C; *R*_f_ 0.76; ^1^H NMR (DMSO-d_6_) (*δ*): 6.47 (s, 1H, CH), 7.32–7.79 (m, 11H, ArH), 8.16–8.18 (m, 2H, ArH). Anal. for (C_22_H_14_O_4_): C 77.18%, H 4.12%; Found: C 77.28%, H 4.22%.

### General synthesis for 8-acetyl-7-hydroxy-4-phenyl-2H-chromen-2-one (10) and 7-hydroxy-4-phenyl-8-propanoyl-2H-chromen-2-one (11)

A mixture of compounds **7** or **8** (1 mmol) and anhydrous AlCl_3_ (5 molar equivalents) was heated to 320 °C and stirred for 2 h at the same temperature. When the reaction mixture was cooled to room temperature, a solution of 5% HCl (30 ml) was added. The resulted suspension was stirred for 1 h at room temperature and then heated under a steam bath for others 30 min. The precipitate formed was filtered off and purified by crystallisation with EtOH to yield compounds **10** and **11**, for which detailed structural assignments are not available in the literature.

**8-Acetyl-7-hydroxy-4-phenyl-2H-chromen-2-one (10)** (CAS Number: 54431-18-4)

Yield: 25%; m.p.: 164–166 °C; *R*_f_ 0.76; ^1^H-NMR (DMSO-d_6_): (*δ*) 1.10 (s, 3H, CH_3_), 6.14 (s, 1H, CH), 6.89 (d, *J* = 8.4, 2H, ArH), 7.33 (d, *J* = 8.4, 2H, ArH), 7.51–7.56 (m, 5H, ArH). ^13^C NMR (126 MHz) (*δ*): 200.37, 159.16, 159.00, 155.37, 151.98, 134.85, 129.62, 129.48, 128.80, 128.33, 115.99, 113.26, 110.65, 39.52, 32.35. Anal. for (C_17_H_12_O_4_): C 72.85%, H 4.32%; Found: C 72.83%, H 4.20%.

**7-Hydroxy-4-phenyl-8-propanoyl-2H-chromen-2-one (11)**

Yield: 20%; m.p.: 172–174 °C; *R*_f_ 0.80. ^1^H NMR (DMSO-d_6_): 1.10 (t, *J* = 7.3, 3H, CH_3_), 2.90 (q, *J* = 7.3, 2H, CH_2_), 6.14 (s, 1H, CH), 6.86 (d, *J* = 8.4, 2H, ArH), 7.31 (d, J = 8.4, 2H, ArH), 7.50–7.55 (m, 5H, ArH). Anal. for (C_18_H_14_O_4_): C 73.46%, H 4.79%. Found: C 73.56%, H 4.99%.

### General synthesis for 8-chloro-7-hydroxy-4-phenyl-2H-chromen-2-one (15) and 8-chloro-7-hydroxy-4-(4-nitrophenyl)-2H-chromen-2-one (16)

To an ice-cold mixture of 2-chlororesorcinol (**14**, 1 mmol) and appropriate ethyl aroylacetate derivatives (**13**, 1 mmol), 96% w/v sulphuric acid (2 ml) was added dropwise. The mixture was brought to room temperature and stirred at 350 rpm by a stirring magnet bar for 24 h, then TLC showed the disappearance of both starting materials. The reaction mixture was quenched with crushed ice flakes, subsequently diluted with H_2_O (10 ml) and extracted with EtOAc (3 × 10 ml). The organic layer was dried with Na_2_SO_4_ and concentrated until dryness under reduced pressure. The targeted compounds **15** or **16** were isolated from the crude by crystallisation with EtOH. The structural assignments of compound **15** were in good agreement with the literature^36^.

**8-Chloro-7-hydroxy-4-phenyl-2H-chromen-2-one (15)** (CAS Number: 53391-82-5)

Yield: 50%; m.p.: 214-215 °C; R*_f_* 0.63; ^1^H-NMR (DMSO-*d*_6_) (δ): 5.89 (s, 1H, CH), 6.66 (d, *J* = 8.8, 1H, ArH), 6.95 (d, *J* = 8.8, 1H, ArH), 7.15–7.25 (m, 5H, ArH). Anal. for (C_15_H_9_ClO_3_): C 66.04%, H 3.33%; Found: C 66.14%, H 3.42%.

**8-Chloro-7-hydroxy-4-(4-nitrophenyl)-2H-chromen-2-one (16)**

Yield: 55%; m.p.: 303–305 °C; *R*_f_ 0.29; ^1^H-NMR (DMSO-d_6_) (*δ*): 6.38 (s, 1H, CH), 6.98 (d, *J* = 8.7, 1H, ArH), 7.15 (d, *J* = 8.7, 1H, ArH), 7.82 (d, *J* = 8.2, 2H, ArH), 8.39 (d, *J* = 8.2, 2H, ArH). ^13^C NMR (DMSO-d_6_) (*δ*): 111.55, 112.08. 113.36, 124.32, 125.99, 130.60, 141.74, 146.71. 148.54, 151.42, 153.76, 157.83, 159.46. Anal. (C_15_H_8_ClNO_5_): C 56.71%, H 2.54%, N 4.41%; Found: C 56.75%, H 2.27%, N 4.44%.

### Synthesis of 4-(4-aminophenyl)-8-chloro-7-hydroxy-2H-chromen-2-one (17)

To a suspension of the nitro derivative **16** (1 mmol) and a catalytic amount of Pd/C in EtOH (15 ml), hydrazine hydrate (NH_2_–NH_2_·H_2_O, 10 molar equivalents) was slowly added. The reaction was stirred and refluxed (70 °C) under a nitrogen atmosphere for 1 h. Then, the mixture was filtered through celite cake, which was later washed with EtOAc. The obtained solution was evaporated *in vacuo* to give the crude product, then diluted with EtOAc and washed with H_2_O (3 × 10 ml). The organic layer was dried with Na_2_SO_4_ and concentrated until dryness. The residue was purified by crystallization with EtOH to give the corresponding amino derivative **17**.

Yield: 40%; m.p.: 313–315 °C; *R*_f_ 0.14; ^1^H-NMR (DMSO-d_6_) (*δ*): 5.65 (bs, 2H, NH_2_), 6.06 (s, 1H, CH), 6.67 (d, *J* = 8.2, 2H, ArH), 6.94 (m, 1H, ArH), 7.21 (d, *J* = 8.2, 2H, ArH), 7.47 (m, 1H, ArH). Anal. for (C_15_H_10_ClNO_3_):C 62.62%; H 3.50%; N 4.87%; Found: C 62.60%, H 3.68%, N 4.65%.

### CA inhibitory assay

An applied photophysics stopped-flow instrument has been used for assaying the CA catalysed CO_2_ hydration activity. Phenol red (at a concentration of 0.2 mM) has been used as an indicator, working at the absorbance maximum of 557 nm, with 10–20 mM Hepes (pH 7.5) or Tris (pH 8.3) as buffers, and 20 mM Na_2_SO_4_ or 20 mM NaClO_4_ (for maintaining constant the ionic strength), following the initial rates of the CA-catalysed CO_2_ hydration reaction for a period of 10–100 s. The CO_2_ concentrations ranged from 1.7 to 17 mM for the determination of the kinetic parameters and inhibition constants. For each inhibitor, at least six traces of the initial 5-10% of the reaction have been used for determining the initial velocity. The uncatalyzed rates were determined in the same manner and subtracted from the total observed rates. Stock solutions of inhibitor (10 mM) were prepared in distilled-deionized water and dilutions up to 0.01 nM were done thereafter with distilled-deionized water. Inhibitor and enzyme solutions were preincubated together for 15 min at room temperature prior to assay to allow for the formation of the E–I complex. The inhibition constants were obtained by non-linear least-squares methods using PRISM 3, as reported earlier and represent the mean from at least three different determinations. CA isoforms were recombinant ones obtained as reported earlier by this group^37–40^.

### Docking studies

Automated docking was carried out by means of the programme AUTODOCK 4.2^41^. The crystal structure of was retrieved from the RCSB Protein Data Bank (PDB: 1JCZ)^42^. The ligand and water molecules were discarded, and hydrogen atoms were added to protein with Discovery Studio 2.5.5. Structures of the ligands were constructed using Discovery Studio 2.5.5 and energy was minimised using the Powel protocol (1000 steps). The regions of interest used by AUTODOCK were defined by considering the suitable ligand docked into the hCA XII receptor as the central group; the docking box included the canonical binding site found for several hydrolysed coumarins; in particular, a grid of 60, 60, and 60 points in the x, y, and z directions was constructed centred on the centre of the mass of metal as Zn^2+^ ion. A grid spacing of 0.375 Å and a distance-dependent function of the dielectric constant were used for the energetic map calculations. Using the Lamarckian Genetic Algorithm (LGA), all docked compounds were subjected to 100 runs of the AUTODOCK search, in which the default values of the other parameters were used. Cluster analysis was performed on the docked results using an RMS tolerance of 2.0 Å. The Lamarckian genetic algorithm was applied to model the interaction between ligands and hCA XII active site. For the Lamarckian genetic algorithm: 27,000 maximum generations; a gene mutation rate of 0.02 and; a crossover rate of 0.8 were used. Cluster analysis was performed on the docked results using an RMSD (Root Mean Square Deviation) tolerance of 2 Å. All the compounds were docked according to the afore mentioned parameters. The hCA XII/ligand complex obtained by docking studies was minimised using 1000 iterations of SD and 1000 interaction of Polak-Ribiere Conjugate Gradient. Interactions were identified using the LigPlot software^43^ and the figures were prepared using opensource software PyMOL (https://pymol.org/2/).

## Results and discussion

**Scheme 1.**
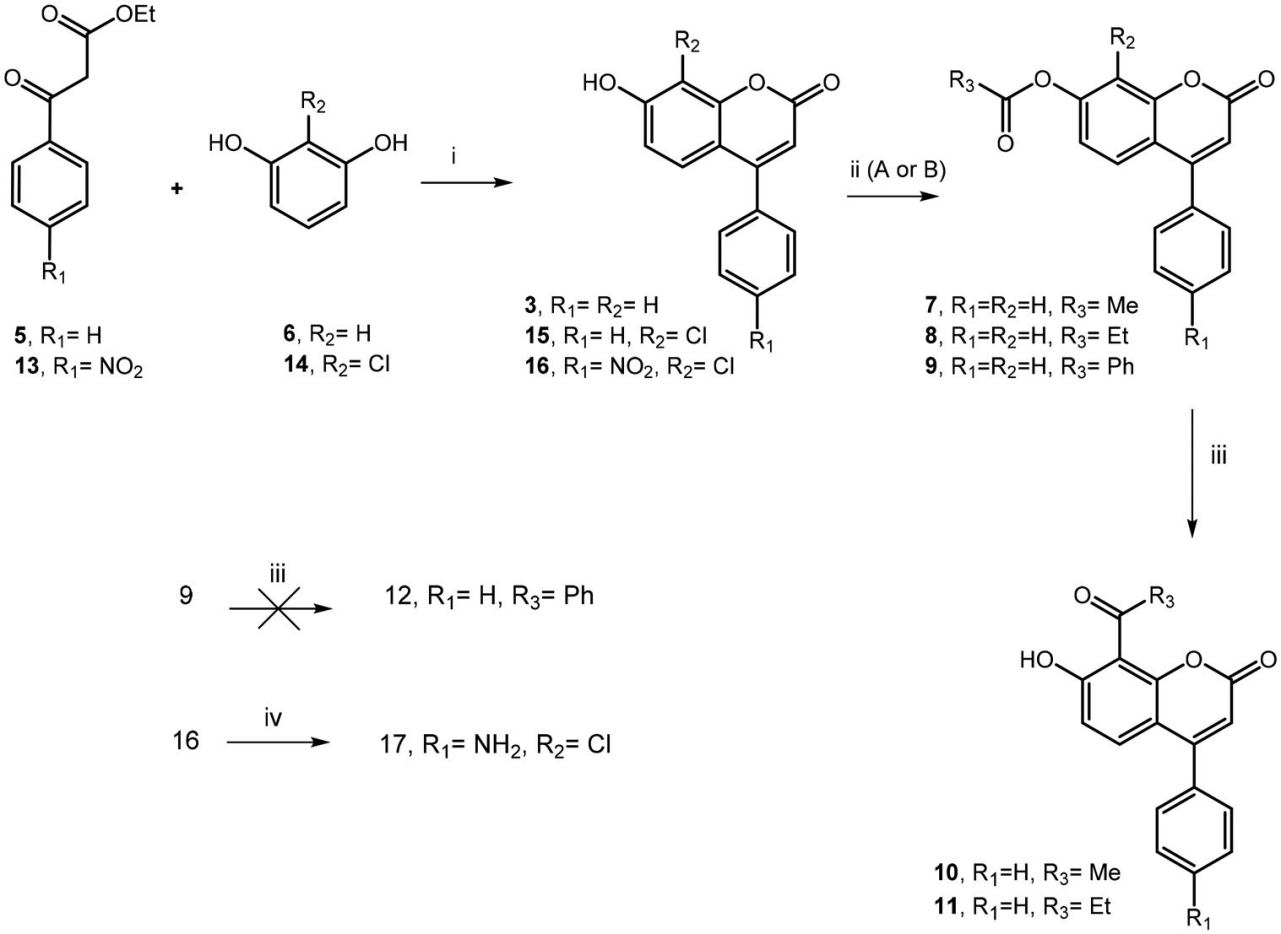
(i) H_2_SO_4_, 0 °C to rt, 18–22 h; (ii) A: (MeCO)_2_O, H_2_SO_4_, Et_3_N, 0 °C to rt, 2 h; B: PheCOCl or EtCOCl, Et_3_N, DCM, rt, 24 h; (iii) AlCl_3,_ 320 °C, 6 h; (iv) NH_2_NH_2_-H_2_O, Pd/C, EtOH, 70 °C, 1 h.

To improve our knowledge on coumarin-based inhibitors from synthetic sources and to study in depth their structure–affinity relationships, we focussed our interest in the substitution pattern at the C7 and C8 position of chromen-2-one nucleus (see Figure 2) of prototypes 4-phenyl-coumarin derivatives **3** and **4** displayed in Figure 2. Firstly, we investigated the role of hydroxyl moiety located in the C-7 position of the coumarin system and synthesised the corresponding acetate **7**, propanoate **8** and benzoate **9** by the reaction of acetic anhydride (pathway A) or suitable chlorides (pathway B) with parent compound **3**, which was prepared in good yields through Pechmann reaction (Scheme 1)^44^ from resorcinol (**6**) and ethyl benzoylacetate (**5**). The obtained esters **7** and **8** were further subjected to Fries rearrangement reaction conditions by employing as Lewis acid AlCl_3_, thus giving the desired **10** and **11** derivatives bearing 8-acetyl or 8-propionyl substituent, respectively. Unfortunately, we failed in all attempts to obtain 8-benzoyl derivative **12** from corresponding benzoate **9**. Moreover, the Pechmann reaction allowed us to prepare further 4-arylcoumarin analogs **15-16**, thus introducing in 8-position a chlorine atom in place of the hydrogen atom. As shown in Scheme 1, the reaction of 2-chlorobenzene-1,3-diol (**14**) with the suitable ethyl benzoylacetate (**5**) or ethyl 3-(4-nitrophenyl)-3-oxopropanoate (**13**) gave corresponding coumarins **15-16**. In turn, by nitroreduction of compound **16,** we prepared the desired 4-aminophenylderivative **17** as the analog of prototype **4**.

### Carbonic anhydrase inhibition

This small series of coumarin derivatives **7–11**, **15–17** were subjected to the stopped-flow CO_2_ hydrase assays to evaluate the binding affinity against the tumour-associated hCA IX and XII over ubiquitous isoforms hCA I and II. The data were collected in Table 1 in comparison with previously reported coumarins **3** and **4** as well as umbelliferon. All tested compounds **7–11** and **15–16** displayed inhibitory effects towards hCA IX and XII with *K*_i_ values in the wide range of 21.8–3042 nM; whereas, these compounds did not inhibit off-target isoforms hCA I and II up to the 10,000 nM concentration in a good agreement with previously reported coumarins **3**–**4** and umbelliferon.

**Table 1.** Inhibition data for hCA I, II, IX and XII with compounds **7–11, 15** and **17** as well as umbelliferon and **3–4** as reference compounds^a^.

	R_1_	R_2_	R_3_	hCA I	hCA II	hCA IX	hCA XII
**Umbelliferon^b^**				>10,000	>10,000	24.9	45.1
**3^b^**	H	H	OH	>10,000	>10,000	47.2	90.8
**4 ^b^**	Me	NH_2_	OH	>10,000	>10,000	24.2	5.5
**7**	H	H	OCOMe	>10,000	>10,000	56.2	734.1
**8**	H	H	OCOEt	>10,000	>10,000	97.8	523.5
**9**	H	H	OCOPh	>10,000	>10,000	21.8	384.6
**10**	COMe	H	OH	>10,000	>10,000	176.5	24.9
**11**	COEt	H	OH	>10,000	>10,000	3042	868.5
**15**	Cl	H	OH	>10,000	>10,000	261.3	32.0
**17**	Cl	NH_2_	OH	>10,000	>10,000	180.3	29.3

^a^Errors in the range of ± 10% of the reported value, from 3 different assays.

^b^Data from reference 29.

A preliminary analysis of structure-affinity-relationship (SAR) for the esters **7**–**9** suggested that the presence of acetyl, propionyl or benzoyl group (e.g. R_3_CO), which masks the hydroxyl moiety of the lead compound **3**, gave an opposite kinetic behaviour against hCA IX and XII. Specifically, the lack of the 7-hydroxyl group did not significantly affect the inhibition potency of benzoate **9** against hCA IX so that its *K*_i_ value 21.8 nM was lower than that of parent compound **3** (*K*_i_ = 47.2 nM) and comparable to umbelliferon (*K*_i_ = 24.9 nM). For acetate **7** and propionate **8,** we found less inhibitory effects towards hCA IX when compared with corresponding hydroxyl derivative **3**. Furthermore, all tested esters **7–9** were up to 8-fold less active towards hCA XII when compared with parent compound **3**. Thus, we can hypothesise that the 7-hydroxyl polar group might play a crucial role in the binding recognition process within hCA XII. Moreover, the *K*_i_ values measured for compounds **10**, **11** and **15** highlighted that the introduction of MeCO- or EtCO-moiety as well as a chlorine atom at the C8 position of coumarin scaffold was detrimental for the binding to hCA IX isoform. On the contrary, compounds **10** and **15** displayed an improved inhibitory effect towards hCA XII when compared to unsubstituted parent compound **3**, whereas as outlier the 7-hydroxy-4-phenyl-8-propanoyl-2*H*-chromen-2-one (**11**) was 10-fold less efficient than prototype **3**. Finally, in the case of compound 4-aminophenyl substituted compound **17**, we found that the presence of a chlorine atom in place of methyl-substituent (i.e. compound **4**) significantly reduced the *K*_i_ affinity, especially towards hCA IX isoform. Overall, coumarin **9** demonstrated the best inhibitory effects and selectivity towards hCA IX; whereas, compounds **10**, **15** and **17** were efficacious hCA XII inhibitors having significant potency and selectivity when compared with umbelliferon.

### Docking studies

**Figure 3.**
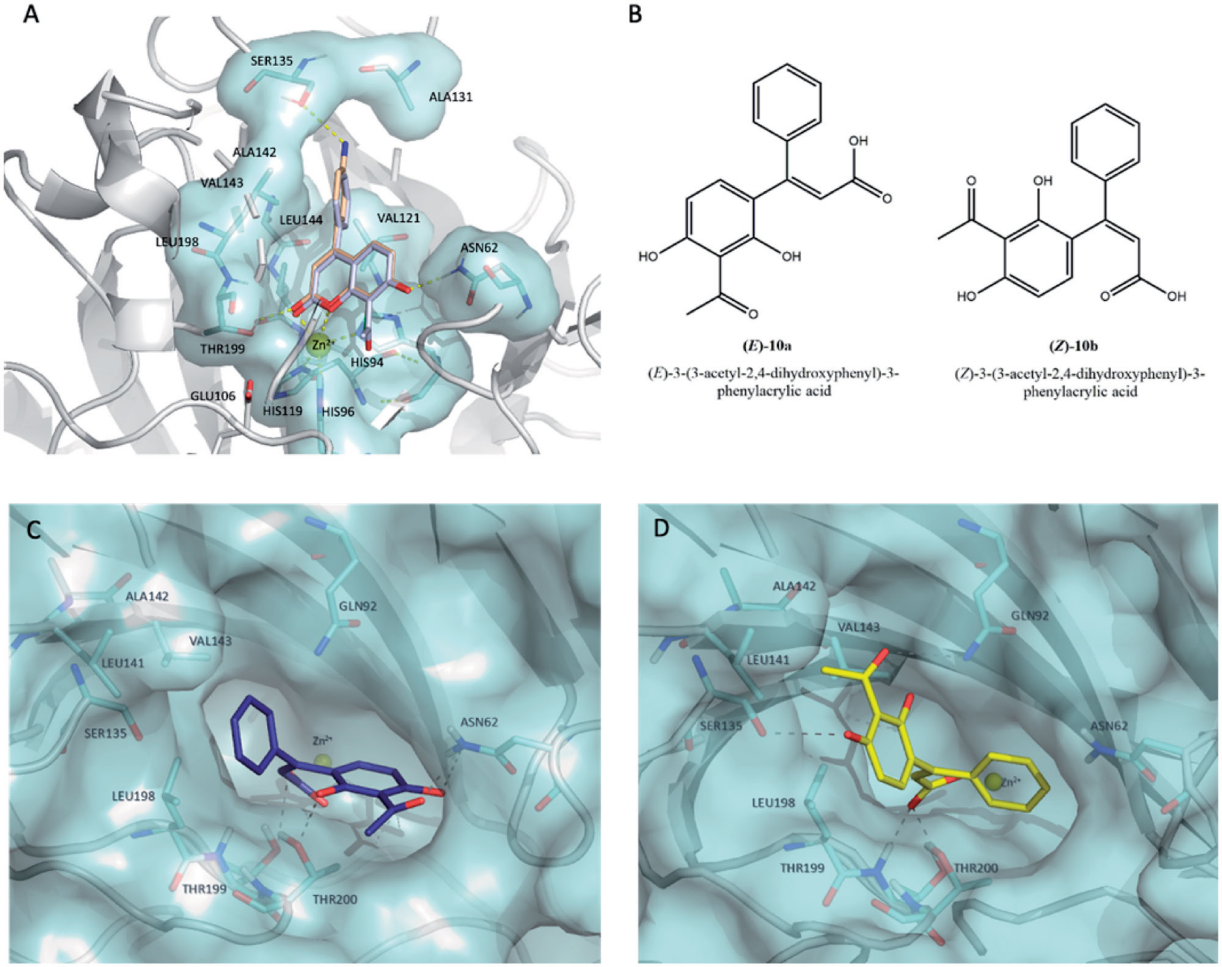
(A) Predicted binding mode of compound **10** (coloured in light blue) overlaid with compound **17** (coloured in wheat) into hCA XII cleft (PDB code 1JCZ).^42^ (B) Chemical structures of hydrolytic forms **10a** and **10b**. Predicted binding mode of (*E*)-**10a** (C, coloured in blue) and (*Z*)-**10b** (D, coloured in yellow**)** as hydrolysed forms of coumarin **10** into hCA XII cleft (PDB Code: 1JCZ)^42^. Compounds and crucial residues are shown as sticks; dashed lines represent hydrogen bond interactions. The protein structure is shown as pale-cyan surface and light grey cartoons. Zinc ion is depicted as a yellow sphere. Figures made by Pymol (https://pymol.org).

In silico studies were carried out in order to elucidate the hypothetical binding pose into the hCA XII catalytic cleft (PDB code 1JCZ)^42^ for the selected coumarins **10** (*K*_i_ value of 24.9 nM) and **17** (*K*_i_ value of 29.3 nM) displaying higher affinity than that of parent compound umbelliferon (*K*_i_ value of 45.1 nM). A previously validated protocol^29^ was applied to perform docking simulations for the newer coumarins studied in this work considering the coumarin system and its hydrolysed products. Firstly, we considered the binding mode of closed forms of inhibitors **10** and **17**; Figure 3 displays their ability to deeply occupy the middle area of the catalytic cavity of hCA XII through two hydrogen bond contacts established by 7-hydroxyl substituent and carbonyl group with hydrophilic residues Asn62 and Thr199, respectively. As you can see in Figure 3(A) the 4-phenyl-substituent is projected towards hydrophobic half of the hCA XII cavity lined by Val121, Val141, Leu142, Val143, and Leu198. Additionally, compound **17** is predicted to bind residue Ser135 by means of H-bond contact mediated by aminophenyl moiety; by comparing the *K*_i_ values of coumarins **10** and **17** we hypothesised that the contact with Ser135 is not crucial to improve the hCA XII affinity. This hypothesis was corroborated by comparing the *K*_i_ value measured for compound **15** for which the amino group is not present. Interestingly, the two studied coumarin **10** and **17** oriented the lactone moiety towards the Zn^2+^ ion, creating a favourable condition for the hydrolysis. Keeping in mind these hypothetical binding modes, we can speculate that these selective inhibitors might be not capable of accessing at the active site of hCA II, that is characterised by the presence of Phe131 residue as the gatekeeper of enzymatic cleft; in the case of hCA IX or hCA XII isoforms, the residues Val131 or Ala131 replace the bulkier Phe131, respectively, thus explaining the hCA IX/hCA XII selectivity of tested coumarins **7-11**, **15-16** over hCA II as found for other coumarin derivatives reported in the literature^30^^,^^45^. This issue could justify the advantageous selectivity profile showed by this class of coumarins that are lacking affinity against off-target isoforms hCA II (*K*_i_ values> 10,000 nM).

Considering that the Zn^2+^–water-activated species can hydrolyse the lactone ring of the benzopyrone system of tested coumarins, we decided to compare the binding pose of selected coumarin **10** with corresponding hydrolysed *cis*-/*trans*-hydroxycinnamic acids (**10a** and **10 b**, Figure 3(B)) that might be derived by hCA esterase activity. Figures 3(C) and 3(D) show the predicted binding mode for the two isomers (*E)*-**10a** and (*Z)-***10b** into hCA XII cleft. Docking simulations suggested that the isomer (*E*)-**10a** (coloured in blue in Figure 3(C)) assumes a similar orientation of parent coumarin **10**. Indeed, the phenyl ring is orientated in the proximity of the hydrophobic residue Leu198 located in the middle area of hCA XII cleft; whereas the 2,4-dihydroxyl phenyl ring is leaned towards the entrance of the active site close to the hydrophobic wall, it engages a dense network of hydrogen bond interactions by C4-hydroxyl and acetyl moiety with Asn62 and C2-hydroxyl group with Thr200. In contrast, the isomer (*Z)-***10b** (coloured in yellow in Figure 3(D)) anchors the hCA XII cavity through H-bond interactions with polar residues Gln92, Ser135, and Thr199 and it assumes a different orientation even if it is equally capable of occluding the entrance of the active site.

In conclusion, we identified a new series of coumarins structurally related to umbelliferon possessing a high affinity towards tumour-expressed hCA IX/XII and selectivity over hCA I/II isoforms. The prediction of binding poses of some inhibitors suggested that this series of compounds might anchor the residues outside of the active site, so that these coumarins belong to non-classical CAIs. Overall, this work might contribute to elucidate the mode of interaction of coumarins against tumour-expressed hCA IX/XII isoforms.
